# Change in treatment burden among people with multimorbidity: Protocol of a follow up survey and development of efficient measurement tools for primary care

**DOI:** 10.1371/journal.pone.0260228

**Published:** 2021-11-29

**Authors:** Hilda O. Hounkpatin, Paul Roderick, James E. Morris, Scott Harris, Forbes Watson, Hajira Dambha-Miller, Helen Roberts, Bronagh Walsh, Dianna Smith, Simon D. S. Fraser, on behalf of the ARC Wessex Treatment Burden Group

**Affiliations:** 1 School of Primary Care, Population Sciences, and Medical Education, University of Southampton, Southampton, United Kingdom; 2 NHS Dorset Clinical Commissioning Group, Dorset, United Kingdom; 3 Human Development and Health, University of Southampton, Southampton, United Kingdom; 4 Geriatric Medicine, University Hospitals Southampton, Southampton, United Kingdom; 5 Health Sciences, University of Southampton, Southampton, United Kingdom; 6 Geography and Environmental Science, University of Southampton, Southampton, United Kingdom; Faculty of Health Sciences - Universidade da Beira Interior, PORTUGAL

## Abstract

**Background:**

Treatment burden is the effort required of patients to look after their health and the impact this has on their functioning and wellbeing. It is likely treatment burden changes over time as circumstances change for patients and health services. However, there are a lack of population-level studies of treatment burden change and factors associated with this change over time. Furthermore, there are currently no practical screening tools for treatment burden in time-pressured clinical settings or at population level.

**Methods and analysis:**

This is a three-year follow-up of a cross-sectional survey of 723 people with multimorbidity (defined as three or more long-term conditions; LTCs) registered at GP practices in in Dorset, England. The survey will repeat collection of information on treatment burden (using the 10-item Multimorbidity Treatment Burden Questionnaire (MTBQ) and a novel single-item screening tool), sociodemographics, medications, LTCs, health literacy and financial resource, as at baseline. Descriptive statistics will be used to compare change in treatment burden since the baseline survey in 2019 and associations of treatment burden change will be assessed using regression methods. Diagnostic test accuracy metrics will be used to evaluate the single-item treatment burden screening tool using the MTBQ as the gold-standard. Routine primary care data (including demographics, medications, LTCs, and healthcare usage data) will be extracted from medical records for consenting participants. A forward-stepwise, likelihood-ratio logistic regression model building approach will be employed in order to assess the utility of routine data metrics in quantifying treatment burden in comparison to self-reported treatment burden using the MTBQ.

**Impact:**

To the authors’ knowledge, this will be the first study investigating longitudinal aspects of treatment burden. Findings will improve understanding of the extent to which treatment burden changes over time for people with multimorbidity and factors contributing to this change, as well as allowing better identification of people at risk of high treatment burden.

## Introduction

Treatment burden is the effort required of patients to look after their health and the impact this has on their functioning and wellbeing [1–4]. Taking and managing multiple medications, organising and attending healthcare appointments, monitoring health, performing self-care, and modifying lifestyle behaviours all contribute to this workload [3]. In addition, patients may experience adverse effects of medications, drug-drug interactions and drug-comorbidity interactions [5]. The need to assess treatment burden in the context of multimorbidity (defined in this study as the presence of three or more long-term conditions) was recognised in the National Institute for Health and Care Excellence (NICE) 2016 guidance for the assessment and management of multimorbidity [6]. People with few chronic conditions, whose treatment is straightforward, may have a treatment workload that is justifiable and well tolerated. For those with greater clinical complexity and fewer available resources, total workload of a treatment regimen might represent a substantial burden, outweighing patient ‘capacity’ (ability to manage workload conferred by both treatment and the demands of everyday life) and risk treatment failure [7].

Treatment burden may be exacerbated by the predominance of single-disease-focused clinical guidelines, with patients often asked to follow multiple treatment plans in parallel [8]. Patients with complex disease and/or multimorbidity may feel overburdened, resulting in low treatment adherence, worse health outcomes and poor quality of life [9–13]. This, in turn, may contribute to health service inefficiency and ineffectiveness, healthcare inequity, and has led to calls for health services to be organised in ways that are as straightforward and holistic as possible for patients and carers, such that care is prioritised from the patient perspective [14, 15]. This is important given the ageing and increasingly multimorbid UK population [16]. Our previous cross sectional study of treatment burden among older people (≥55years) with multimorbidity (defined as having three or more long-term conditions from a specified list) [17] in the county of Dorset, England found that approximately one in five people experience high treatment burden and that high treatment burden was associated with limited health literacy, financial difficulty, having more long-term conditions and greater number of prescribed regular medications [18]. However, for commissioners and those planning service delivery for people with complex needs, no population-level longitudinal studies exist that give an indication of the extent to which treatment burden changes over time. In addition, little is known about the factors (both individual and system-driven) associated with change in treatment burden over time. At health system level, interventions that reduce treatment burden and foster ‘minimally disruptive medicine’ may lead to efficiencies and improve quality of care as well as improving patients’ quality of life [19]. Healthcare systems will also need to consider how to identify people at risk of being overburdened [20]. At the individual patient-clinician level, assessing the degree of treatment burden allows for discussion with patients about optimising treatments and care,–balancing the management priorities of different conditions, and exploring if total healthcare workload is compatible with patients’ daily lives. Several quantitative measures of treatment burden have been developed and validated [21–28]. However, routinely measuring treatment burden using current questionnaire measures is not practical in most clinical settings, even employing those tools with fewer items [22]. At system level, being able to identify those at risk of being overburdened via routinely collected data would assist with care planning.

This protocol describes a follow-up survey study of 723 people aged 55+ years with multimorbidity in Dorset, England, who took part in the baseline survey and consented to complete a follow-up survey within three years. The baseline survey was conducted between February and July 2019, prior to the COVID-19 pandemic [18]. Dorset health and social care provision underwent a large-scale Clinical Services Review prior to 2019 and reconfiguration of services delivering care to people with LTCs has been implemented since the baseline survey [29]. This follow-up survey is expected to be conducted between August and October 2021.

## Materials and methods

### Aims and objectives

This follow up cross-sectional survey of people with multimorbidity will quantify change in treatment burden over time and evaluate factors associated with treatment burden change. The survey will also facilitate evaluation of the validity of a single-item treatment burden question, modified from an item originally trialled in the baseline survey. Survey-measured, self-reported treatment burden will be compared to routine primary care metrics and the feasibility of developing a risk tool for high treatment burden based on routine data will be explored. The protocol described in this peer-reviewed article is published on protocols.io, https://dx.doi.org/10.17504/protocols.io.bw5fpg3n and is included for printing as S1 File with this article.

The objectives of the study are to:

1. Explore the degree to which treatment burden changes over time and the factors associated with such change.
2. Evaluate the validity of a single-item treatment burden screening tool versus high treatment burden at follow-up.
3. Undertake descriptive comparison of survey-measured, self-reported treatment burden with relevant metrics extracted from the primary care records of surveyed patients who provide consent for this aspect.
4. Develop and test the internal validity of a high treatment burden predictive risk tool for use in primary care/commissioning settings to identify those at risk of high treatment burden using metrics from routine clinical data of those surveyed, in comparison with survey-measured self-reported treatment burden. If secondary care data are available, then use this to further enhance the risk tool.
5. Explore whether there is any relationship between our measured change in treatment burden and the reconfiguration of services in Dorset.

### Ethics

This study protocol has received Research Ethics Committee approval (reference: 21/EM/0021) and Health Research Authority (HRA) approval (IRAS project ID: 292906) on 27 January 2021.

### Study sites and participants

All eight GP practices who took part in the baseline study will be approached to assist with this follow-up study. These practices were selected to represent a mixture of urban and rural, affluent and more deprived locations across Dorset. A flowchart of the methods and analysis is presented in S1 Fig.

Study participants will be patients within these practices who took part in the baseline survey and consented to receiving the follow up survey (n = 723). The original inclusion criteria of the baseline survey were those aged 55 years or over and living with at least three of the following LTCs: atrial fibrillation, coronary heart disease, heart failure, hypertension, peripheral arterial disease, stroke or transient ischaemic attack, diabetes, asthma, chronic obstructive pulmonary disease, depression, chronic kidney disease, epilepsy, osteoporosis, rheumatoid arthritis, Parkinson’s disease, multiple sclerosis, inflammatory bowel disease, coeliac disease, osteoarthritis. We defined multimorbidity as three or more chronic conditions for the following reasons: (i) to be consistent with the baseline study, (ii) the sample size calculation was based on the prevalence of a previous study which used this definition, and (iii) this has been considered to be a more valid cut-off score than the criterion of two chronic conditions for multimorbidity in elderly patients [30]. The specified diseases were selected as they are common, readily identified from GP records, and represented a range of body systems. Diseases were defined using the Quality and Outcome Framework (QoF) clinical code and Read codes [22, 23, 31, 32]. Only one person from a given household was eligible to participate [18]. Exclusion criteria were: those living in a care home, receiving palliative care, presence of a mental health diagnosis (psychosis, schizophrenia, bipolar disorder) or dementia, active cancer (recorded in the last three years), an expressed wish not to participate in research, lack of capacity to participate in the study, or any other reason at the discretion of healthcare professionals at the GP practice with sufficient knowledge of the patient which renders the patient unsuitable to receive the survey (for example if they were very frail, having cancer investigations, had a very recent bereavement It was not considered ethically appropriate or acceptable to send the survey out to patients who met the exclusion criteria as they might find it particularly distressing. Each participating site applied the same algorithm to electronically search and identify potential participants. Manual screening was performed by a health professional to exclude patients that met the exclusion criteria. A maximum of 250 patients (randomly selected from all potentially eligible participants) from each practice were invited to participate in the baseline survey.

### Participant recruitment and invitation

Participating practices will be asked to identify the patients who were participants in the baseline survey and consented to receiving the follow-up survey, using a list of unique study IDs. Lists of these potentially eligible patients will be manually screened by GPs (or another suitable healthcare professional with sufficient patient knowledge from the GP practice) for exclusion criteria (as some participants may now meet the exclusion criteria). Practices will be encouraged to only exclude people if they feel it is absolutely necessary, while acknowledging that in some circumstances, sending the follow-up survey may be inappropriate.

#### Survey pack mail out

The research team will prepare survey packs (containing a participant information sheet, carbon duplicate consent form, survey booklet, two pre-addressed freepost return envelopes, and a £5 gift voucher as an incentive), having affixed study ID labels to survey and consent forms. These prepared survey packs will be supplied to GP practices, who at the point of invitation will: (i) add a prepared covering letter to the pack (headed up and signed by the GP practice), personalising the letter with the invitee’s name and address; (ii) add the invitee’s name and address to the outer envelope of the survey pack. Patient details and study IDs will be matched in accordance with practices’ research records. Practices will post out survey packs on behalf of the research team. Recipients will be asked to complete the paper consent form and paper survey booklet and return both by post in the separate envelopes provided within one month of receiving the survey pack. An online response platform is not being provided for this follow-up survey as a result of the very low uptake of this response option (3.2%) observed in the baseline survey.

### Survey items

For this follow-up survey, changes from the baseline survey design are minimal in order to provide maximally comparable follow-up data from that acquired at baseline. Survey questions and response options of the baseline survey are available elsewhere [18].

#### Outcomes

The survey will measure self-reported treatment burden using the 10-item Multimorbidity Treatment Burden Questionnaire (MTBQ [22]. The MTBQ is short, simple, and easy to use and has been validated in a multimorbid population similar to that in the current study. It has demonstrated good validity, reliability and responsiveness. Scores for each MTBQ item range from 0 (not difficult/does not apply) to 4 (extremely difficult). The average score is multiplied by 25 to yield a score ranging from 0 to 100 and then categorised to non (global score of 0), low (>0 and <10), medium (≥10 and <22), or high (≥22). A novel single-item screening tool for treatment burden that was developed together with our public contributors will also be included in the survey, in place of the original exploratory single-item measure included in the baseline survey. This novel single-item tool asked: “Have you felt overstretched by everything you’ve had to do to manage your health in the last month (e.g., taking medicines, getting prescriptions, attending appointments)?” to which participants could respond ‘yes’ or ‘no’.

#### Exposures

Prescribed regular medication, number of (and specific) LTCs based on survey inclusion criteria, travel for healthcare, recent healthcare resource use, and health status/quality of life (measured using the Short form-12 Health Survey (SF-12)) will also be collected.

#### Potential confounders

Sociodemographic information including age (as a continuous variable), sex (male/female), marital status (married or in a civil partnership, single (never married or in a civil partnership), divorced or dissolved civil partnership, widowed) were collected in the survey.

#### Potential effect modifiers

Level of education (not asked at baseline) based on the following categories: NVQ4/NVQ5/Degree or equivalent, NVQ3/GCE A Level equivalent/4 NVQ2/GCE O Level equivalent/5NVQ1/CSE other grade equivalent, or no qualification; home ownership (home owner/non-home owner); capacity to manage health such as financial resources [perceived level of difficulty in meeting financial costs of healthcare (on a 5-point Likert scale from ‘no’ to ‘extreme’ difficulty)] and health literacy [assessed using the Single Item Literacy Screener (SILS) which measures perceived frequency of needing help to read health-related written material (on a 5-point Likert scale, where ‘sometimes’, ‘often’ and ‘always’ indicate limited health literacy] were also asked in the survey as they may be expected to modify the relationship between exposures and treatment burden.

### Data extraction from primary care records

Data from primary care records will be extracted for respondents who consent to data from their GP record being shared with the research team. Extracted data will include: demographic details (current age, sex, full postcode) as recorded in primary care records, frailty score (electronic Frailty Index and/or Rockwood score where available), main diagnosed health conditions as defined by Read codes, medications on repeat prescription (including details of dose, frequency, and route of administration), recent healthcare usage data (including number of GP practice appointments by type -surgery appointment, telephone consultation, home visit- and role (GP, practice nurse, or other team member), number of outpatient appointments/referrals to secondary care, accident and emergency attendance (if available) and hospital admission data (a count of discharge summaries), as available in the primary care record. We will discuss with colleagues in the CCG about the potential for enhancing primary care data with data from secondary care. These variables have been selected as they may be expected to be associated with high treatment burden.

#### Minimising potential bias

To reduce selection bias, we invited all patients who took part in the baseline survey and consented to receiving a follow-up survey to take part in the current study. This sample includes participants from a range of practices (urban vs rural) socioeconomic background, geographical location. Furthermore, all participants will be treated the same way (i.e., they will receive the same survey asking same questions/measures and be asked to be returned the survey in same way). The same algorithm will also be used across all practices to extract routine data in order to reduce measurement bias.

### Sample size

The pool of participants is limited to the 723 individuals who consented to receiving the follow-up survey. The sample size of the baseline survey was determined using nQuery based on prevalence of high treatment burden (26.6%) from the validation study of the MTBQ together with estimates of likely response rate of approximately 50% (based on the literature) and distribution of the MTBQ score [18]. It was estimated that a minimal sample size of 150 participants would be needed to detect the minimum possible change in treatment burden score (1 point in the 40-point MTBQ raw score) over time. A follow-up sample of 361 participants is anticipated, assuming a likely follow-up survey response rate response rate of 50%.

#### Missing data

We will provide data on the numbers of people who are lost to follow-up and reasons for loss to follow-up. We will produce a descriptive table of characteristics of participants who took part in the follow-up survey and those who didn’t (based on baseline survey data) and assess if any differences are statistically significant. We will explore the extent of missing data on variables of interest and consider imputation techniques where possible [33].

### Statistical analyses

Descriptive statistics will be used to compare the numbers and proportions of participants reporting ‘none’, ‘low’, ‘medium’ and ‘high’ levels of treatment burden based on the global MTBQ score (score of 0, <10, ≥10, ≥22, respectively). We will consider those who report a ≥1 point change in the 40-point MTBQ raw score as having experienced a change in treatment burden, though we recognise this change may not be clinically meaningful. Change in global score and change in these categories between baseline and follow-up will be compared. Characteristics (such as age, sex, education status, and comorbidities) of participants who experience change in treatment burden, those who experience an increase in treatment burden, those who experience a decrease in treatment burden and those who don’t will be described. We will also describe and compare characteristics of those who have experienced an increase in treatment burden to those who have not and those who have experienced a decrease in treatment burden to those who have not. Normally distributed variables will be compared using t-test and categorical variables will be compared using chi-squared tests. Associations with change in categories of treatment burden (i.e., overall change vs no change; decrease vs no change or increase; increase vs no change or decrease) will be examined using a linear mixed model that additionally adjusts for potential confounders. We will also examine effect modification using interaction terms. We will also explore geographical access aspects of treatment burden, considering issues such as distance and travel time to GP and hospital services and distance /time from pharmacies (and/or whether individuals are registered with dispensing practices). Further consideration will be given to availability of public transportation for any of these services. Distance to services for this population may be contrasted with access to treatment for similar age/sex populations in other areas of Dorset to assess whether there is significantly better/worse service provision for the study population. Regressions will adjust for time (year) to account for (and explore impact of) changes to services in Dorset.

The single-item screening tool (at follow-up) will be evaluated relative to high treatment burden as indicated by the MTBQ (at follow-up) using sensitivity, specificity, positive and negative predictive values, and positive and negative likelihood ratios. Area under the receiver operator characteristic curve will also be assessed.

To develop a population-level high treatment burden risk prediction tool, variables available in routine data (such as number of prescribed medications, number of appointments, number of hospital admissions (if available), number of LTCs) will be included in a first stage of a binary logistic regression model predicting risk of high treatment burden based on the MTBQ measure (a score ≥22 indicating high treatment burden versus a score <22 indicating non-high treatment burden). After assessing extent of missing data, a forward-stepwise, likelihood-ratio model building approach will be employed, with only those variables having a statistically significant (p<0.05) effect on the final model equation remaining at the terminal step [34]. A binary logistic regression model containing only these variables will be fitted and weighted scores based on the beta coefficients derived. Model performance will be determined using the area under the receiver operator characteristic curve (to assess discriminant capacity of the model) and calibration plots (to assess calibration) [34].

Data analyses will be led by a public health researcher (HH) with input from a senior medical statistician (SH). All authors (comprising senior academics, academic GPs, Director of Public Health, Chair of the Clinical Commissioning Group, specialist registrars in public health and elderly care, and public contributors) will be involved in interpretation of analyses and results.

### Mapping changes in primary and care services

Working with leaders of the Clinical Commissioning Group we will identify the key changes in primary and secondary care delivery that have occurred in the intervening period including structural issues (such as infrastructure change and location of services and workforce), process issues (such as change in care pathways, virtual vs. in person clinical encounters) and outputs (such as changes in numbers of patients seen in different settings).

### Patient and public involvement

Two public contributors, both living with multiple LTCs, have been involved in the design of the study and are co-investigators. They have reviewed the lay summary and provided valuable insights into the challenges of living with multiple conditions, including the impact of treatment workload on social care. Both will continue engagement with the project throughout its duration and will contribute to outputs and dissemination.

### Dissemination

Study findings will be published in peer-reviewed academic journals and presented at relevant national and international conferences. We will engage with relevant stakeholders to disseminate findings throughout Dorset. Opportunities to implement a high treatment burden risk tool in practice will be explored.

## Discussion

This study will provide the first longitudinal data on treatment burden in those with multimorbidity. This valuable information on the extent and patterning of treatment burden at scale (i.e. the proportion of people that experience ‘no’, ‘low’, ‘medium’, high’ levels of treatment burden) and how these change over time will support planning and evaluation of system-level interventions aimed at optimising treatment burden. This is particularly pertinent to areas such as Dorset given the high proportion of older people in the population as well as the recent changes implemented in the healthcare system, which seek to improve integration of care in the region. This included the expansion of ‘community hubs’ throughout Dorset which bring together primary and secondary care services, improving convenience for patients in terms of access to healthcare. Changes in treatment burden may therefore be due to either patient-level or system-level factors, or both. Furthermore, the occurrence of the Covid-19 pandemic since 2019 has potentially altered several aspects of access to, and use of, health services for people with multimorbidity.

The study will also enable exploration of factors associated with change in treatment burden. There are no studies that have evaluated factors associated with change in treatment burden as a core research objective; one study has briefly assessed change in treatment burden over a 9-month follow-up period and found that change in treatment burden was associated with changes in quality of life. However, further exploration using longer follow-up periods is needed [21]. Understanding which factors are associated with change in treatment burden is needed to help anticipate patient trajectories and inform future interventions and healthcare delivery, both at the individual and health system level.

A single-item treatment burden screening tool that performs well can help clinicians quickly identify those who may feel overburdened. Similarly, a predictive model of high treatment burden could find direct utility in primary care. For example, this would allow comparison between treatment burden across different populations and systems. This would enable ready identification of those at risk of high treatment burden and targeted efforts aimed at its reduction. Increased recognition of treatment burden through its measurement at scale would also sharpen the focus of doctor-patient interactions to place greater emphasis on the priorities of patients. This can help prevent over-medicalisation and assist clinicians in delivering optimal patient-centred care.

### Strengths and limitations

Strengths of the study include a reasonable likelihood of a high response rate given that participants are those who responded to a similar baseline survey, the inclusion of participants from diverse socioeconomic backgrounds and the use of a validated measure of treatment burden in the survey. However, there are important limitations that should be considered. The first is the potential impact of the COVID-19 pandemic on the follow-up survey. In view of the pandemic, the timing of distribution of the follow-up survey has been delayed until late summer 2021 to minimise any potential impact as well as to allow GP practices more time to engage with the study. Despite this, the pandemic has the potential to influence the survey in a number of ways. Firstly, participants may themselves have had COVID, which might have increased their recent experience of ill-health and treatment burden. Secondly, changes to healthcare delivery as a result of the pandemic may mean that most participants will have had reduced or altered healthcare appointments and use of other healthcare resources, which may influence their responses. This will potentially make comparison to baseline survey responses challenging, particularly for the survey section about healthcare usage but also potentially to the treatment burden and quality of life questions. However, the effect on responses to the MTBQ is likely to be small due to the nature of the MTBQ questions, which ask about current rather than past experiences. Other limitations include the inability to infer causality from any observed associations. It is also possible the screening of consented patients for participation could lead to a sample that is skewed towards the lower end of the treatment burden scale. Finally, a possible limitation is that metrics available in routine data may not sufficiently reflect experiences of treatment burden. This may be particularly true given high treatment burden was associated with markers of capacity (health literacy, financial resource) [17], which are not routinely collected. However, this will be an important part of the exploration of the feasibility of developing a high treatment burden risk tool.

## Supporting information

S1 File Protocol registration. (TIF)

S1 Fig Flow chart of methods and analysis. (TIF)

## References

[1] Mair FS, May CR. Thinking about the burden of treatment. BMJ. 2014;349 doi: 10.1136/bmj.g6680 25385748

[2] Sav A, King MA, Whitty JA, Kendall E, McMillan SS, Kelly F, et al. Burden of treatment for chronic illness: a concept analysis and review of the literature. Health Expect. 2015;18(3):312–24. doi: 10.1111/hex.12046 23363080 PMC5060781

[3] Shippee ND, Shah ND, May CR, Mair FS, Montori VM. Cumulative complexity: a functional, patient-centered model of patient complexity can improve research and practice. J Clin Epidemiol. 2012;65(10):1041–51. doi: 10.1016/j.jclinepi.2012.05.005 22910536

[4] Eton DT, Ramalho de Oliveira D, Egginton JS, Ridgeway JL, Odell L, May CR, et al. Building a measurement framework of burden of treatment in complex patients with chronic conditions: a qualitative study. Patient Relat Outcome Meas. 2012;3:39–49. doi: 10.2147/PROM.S34681 23185121 PMC3506008

[5] Hounkpatin HO, Harris S, Fraser SDS, Day J, Mindell JS, Taal MW, et al. Prevalence of chronic kidney disease in adults in England: comparison of nationally representative cross-sectional surveys from 2003 to 2016. *BMJ Open* 2020;10:e038423 doi: 10.1136/bmjopen-2020-038423 32792448 PMC7430464

[6] National Institute for Health and Care Excellence (NICE). Multimorbidity: clinical assessment and management (NG56). Sept 2016.

[7] May CR, Eton DT, Boehmer K, Gallacher K, Hunt K, MacDonald S, et al. Rethinking the patient: using burden of treatment theory to understand the changing dynamics of illness. BMC Health Serv Res. 2014;14:281. doi: 10.1186/1472-6963-14-281 24969758 PMC4080515

[8] Hughes LD, McMurdo ME, Guthrie B. Guidelines for people not for diseases: the challenges of applying UK clinical guidelines to people with multimorbidity. Age Ageing. 2013;42(1):62–9. doi: 10.1093/ageing/afs100 22910303

[9] Pifferi M, Bush A, Di Cicco M, Pradal U, Ragazzo V, Macchia P, et al. Health-related quality of life and unmet needs in patients with primary ciliary dyskinesia. Eur Respir J. 2010;35(4):787–94. doi: 10.1183/09031936.00051509 19797134

[10] Vijan S, Hayward RA, Ronis DL, Hofer TP. Brief report: the burden of diabetes therapy: implications for the design of effective patient-centered treatment regimens. J Gen Intern Med. 2005;20(5):479–82. doi: 10.1111/j.1525-1497.2005.0117.x 15963177 PMC1490106

[11] Ho PM, Rumsfeld JS, Masoudi FA, McClure DL, Plomondon ME, Steiner JF, et al. Effect of medication nonadherence on hospitalization and mortality among patients with diabetes mellitus. Arch Intern Med. 2006;166(17):1836–41. doi: 10.1001/archinte.166.17.1836 17000939

[12] Rasmussen JN, Chong A, Alter DA. Relationship between adherence to evidence-based pharmacotherapy and long-term mortality after acute myocardial infarction. JAMA. 2007;297(2):177–86. doi: 10.1001/jama.297.2.177 17213401

[13] Demain S, Gonçalves A-C, Areia C, Oliveira R, Marcos AJ, Marques A, et al. Living with, managing and minimising treatment burden in long term conditions: A systematic review of qualitative research. *PLoS ONE*. 2015;10(5):e0125457. doi: 10.1371/journal.pone.0125457 26024379 PMC4449201

[14] May C, Montori VM, Mair FS. We need minimally disruptive medicine. BMJ. 2009 Aug 11;339:b2803. doi: 10.1136/bmj.b2803 19671932

[15] Abu Dabrh AM, Gallacher K, Boehmer KR, Hargraves IG, Mair FS. Minimally disruptive medicine: the evidence and conceptual progress supporting a new era of healthcare. J R Coll Physicians Edinb. 2015;45(2):114–7. doi: 10.4997/JRCPE.2015.205 26181525

[16] Barnett K, Mercer SW, Norbury M, Watt G, Wyke S, Guthrie B. Epidemiology of multimorbidity and implications for health care, research, and medical education: a cross-sectional study. Lancet. 2012;380(9836):37–43. doi: 10.1016/S0140-6736(12)60240-2 22579043

[17] Harrison C, Britt H, Miller G, Henderson J. Examining different measures of multimorbidity, using a large prospective cross-sectional study in Australian general practice. BMJ Open. 2014;4(7):e004694. doi: 10.1136/bmjopen-2013-004694 25015470 PMC4120329

[18] Morris JE, Roderick PJ, Harris S, Yao G, Crowe S, Phillips D, et al. Treatment burden for patients with multimorbidity: cross-sectional study with exploration of a single-item measure. Br J Gen Pract. 2021;71(706):e381–e390. doi: 10.3399/BJGP.2020.0883 33875419 PMC8074644

[19] Browne S, Macdonald S, May C, Macleod U, Mair F. Patient, carer and professional perspectives on barriers and facilitators to quality care in advanced heart failure. PLoS ONE. 2014:9(3):e93288 doi: 10.1371/journal.pone.0093288 24676421 PMC3968134

[20] Shippee ND, Allen SV, Leppin AL, May CR, Montori VM. Attaining minimally disruptive medicine: context, challenges and a roadmap for implementation. J R Coll Physicians Edinb. 2015;45:18–22. doi: 10.4997/JRCPE.2015.206 26181526

[21] Tran VT, Montori VM, Eton DT, Baruch D, Falissard B, Ravaud P. Development and description of measurement properties of an instrument to assess treatment burden among patients with multiple chronic conditions. BMC Med. 2012;10:68. doi: 10.1186/1741-7015-10-68 22762722 PMC3402984

[22] Duncan P, Murphy M, Man MS, Chaplin K, Gaunt D, Salisbury C. Development and validation of the Multimorbidity Treatment Burden Questionnaire (MTBQ). BMJ Open. 2018;8(4):e019413. doi: 10.1136/bmjopen-2017-019413 29654011 PMC5900423

[23] Boyd CM, Wolff JL, Giovannetti E, Reider L, Weiss C, Xue QL, et al. Health care task difficulty among older adults with multimorbidity. Med Care. 2014; 52: S118–S125. doi: 10.1097/MLR.0b013e3182a977da 24561750 PMC3937858

[24] Gibbons CJ, Kenning C, Coventry PA, Bee P, Bundy C, Fisher L, et al. Development of a Multimorbidity Illness Perceptions Scale (MULTIPleS). PLoS One. 2013; 8(12): e81852. doi: 10.1371/journal.pone.0081852 24376504 PMC3869652

[25] Eton DT, Elraiyah TA, Yost KJ, Ridgeway JL, Johnson A, Egginton JS, et al. A systematic review of patient-reported measures of burden of treatment in three chronic diseases. Patient Relat Outcome Meas. 2013; 5;4:7–20. doi: 10.2147/PROM.S44694 23833553 PMC3699294

[26] Eton DT, Ridgeway JL, Egginton JS, Tiedje K, Linzer M, Boehm DH, et al. Finalizing a measurement framework for the burden of treatment in complex patients with chronic conditions. Patient Relat Outcome Meas. 2015;6:117–26. doi: 10.2147/PROM.S78955 25848328 PMC4383147

[27] Eton DT, Yost KJ, Lai JS, Ridgeway JL, Egginton JS, Rosedahl JK, et al. Development and validation of the Patient Experience with Treatment and Self-management (PETS): a patient-reported measure of treatment burden. Qual Life Res. 2017;26(2):489–503. doi: 10.1007/s11136-016-1397-0 27566732 PMC5753596

[28] Rogers EA, Yost KJ, Rosedahl JK, Linzer M, Boehm DH, Thakur A, et al. Validating the Patient Experience with Treatment and Self-Management (PETS), a patient-reported measure of treatment burden, in people with diabetes. Patient Relat Outcome Meas. 2017;8:143–156. doi: 10.2147/PROM.S140851 29184456 PMC5687778

[29] NHS Dorset Clinical Commissioning Group. Clinical Services Review [Available from: https://www.dorsetsvision.nhs.uk/about/csr/].

[30] Schram MT, Frijters D, van de Lisdonck EH, Ploemacher J, de Craen AJM, de Waal MWM, et al. Setting and registry characteristics affect the prevalence and nature of multimotbidity in the elderly. J Clin Epidemiol. 2008, 61: 1104–1112. doi: 10.1016/j.jclinepi.2007.11.021 18538993

[31] NHS Digital. Quality and Outcomes Framework (QOF) business rules v41/v42. https://digital.nhs.uk/data-and-information/data-collections-and-data-sets/data-collections/qualityand-outcomes-framework-qof (accessed 4 Aug 2021).

[32] NHS Digital. Technology Reference data Update Distribution [TRUD]: Read codes. https://isd.digital.nhs.uk/trud3/user/guest/group/0/pack/9 (accessed 4 Aug 2021).

[33] Jakobsen JC, Gluud C, Wetterslev J, Winkel P. When and how should multiple imputation be used for handling missing data in randomised clinical trials—a practical guide with flowcharts. BMC Med Res Methodol 2017;17:162. doi: 10.1186/s12874-017-0442-1 http://www.ncbi.nlm.nih.gov/pubmed/29207961 29207961 PMC5717805

[34] Steyerberg EW. Clinical prediction models: a practical approach to development, validation, and updating. Springer, 2009.

