# Supplementary figures and images for: Change in treatment burden among people with multimorbidity: Protocol of a follow up survey and development of efficient measurement tools for primary care
Source: PLoS One. 2021 Nov 29;16(11):e0260228. doi: 10.1371/journal.pone.0260228 (PMC8629211; doi:10.1371/journal.pone.0260228)

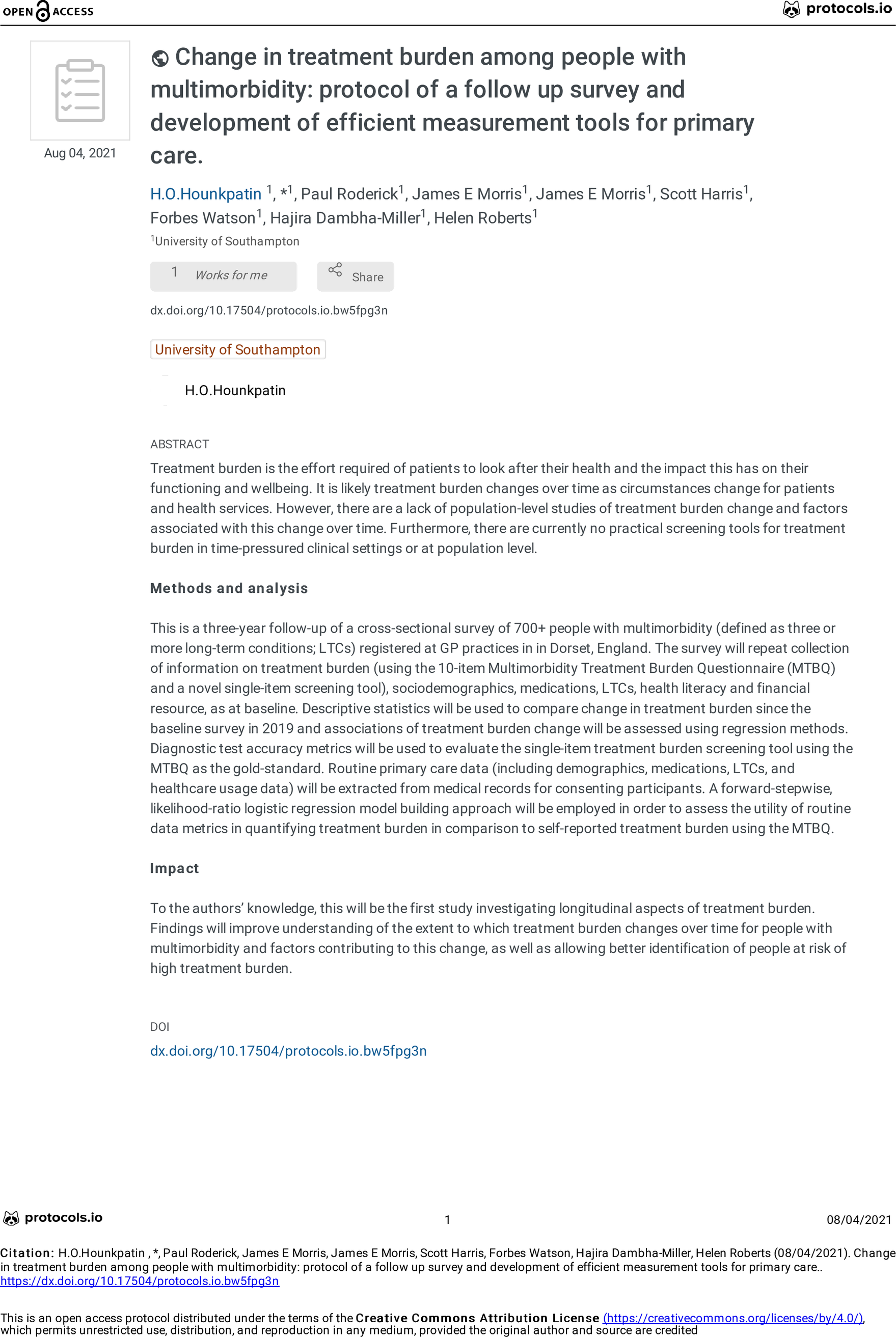

Supplement: S1 File — (TIF) [file pone.0260228.s001.tif]

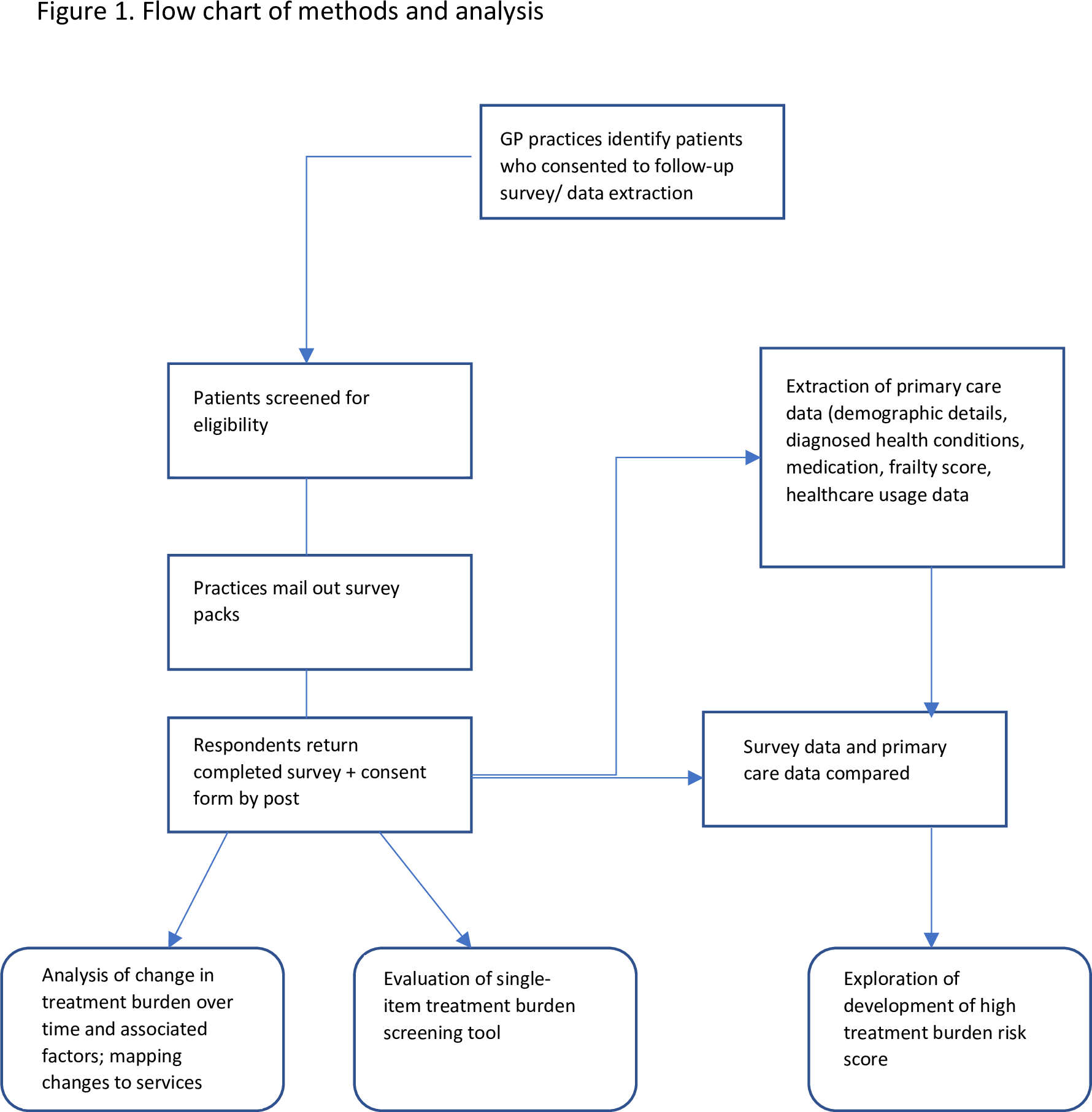

Supplement: S1 Fig — (TIF) [file pone.0260228.s002.tif]
